# DEV induce autophagy via the endoplasmic reticulum stress related unfolded protein response

**DOI:** 10.1371/journal.pone.0189704

**Published:** 2017-12-22

**Authors:** Haichang Yin, Lili Zhao, Xinjie Jiang, Siqi Li, Hong Huo, Hongyan Chen

**Affiliations:** State Key Laboratory of Veterinary Biotechnology, Heilongjiang Provincial Key Laboratory of Laboratory Animal and Comparative Medicine, Harbin Veterinary Research Institute, the Chinese Academy of Agriculture Science, Harbin, P. R. China; University of Hong Kong, HONG KONG

## Abstract

Duck enteritis virus (DEV) can infect ducks, geese, and many other poultry species and leads to acute, septic and highly fatal infectious disease. Autophagy is an evolutionarily ancient pathway that plays an important role in many viral infections. We previously reported that DEV infection induces autophagy for its own benefit, but how this occurs remains unclear. In this study, endoplasmic reticulum (ER) stress was triggered by DEV infection, as demonstrated by the increased expression of the ER stress marker glucose-regulated protein 78 (GRP78) and the dilated morphology of the ER. Pathways associated with the unfolded protein response (UPR), including the PKR-like ER protein kinase (PERK) and inositol-requiring enzyme 1 (IRE1) pathways, but not the activating transcription factor 6 (ATF6) pathway, were activated in DEV-infected duck embryo fibroblast (DEF) cells. In addition, the knockdown of both PERK and IRE1 by small interfering RNAs (siRNAs) reduced the level of LC3-II and viral yields, which suggested that the PERK-eukaryotic initiation factor 2α (eIF2α) and IRE1-x-box protein1 (XBP1) pathways may contribute to DEV-induced autophagy. Collectively, these data offer new insight into the mechanisms of DEV -induced autophagy through activation of the ER stress-related UPR pathway.

## Introduction

Duck enteritis virus (DEV) is a double-stranded, linear DNA virus; with a genome of approximately 150 KB. It belongs to the family Herpesviridae, subfamily Alphaherpesviridae and genus Mardivirus. DEV has a morphology typical of Herpesviruses, with a spherical viral particle that includes the core, capsid, outer membrane and envelopes. In addition, a tegument structure exists between the capsid and the envelopes. The virus can infect a variety of waterfowl causing an acute, septic and highly fatal infectious disease named duck viral enteritis. The symptoms of this infection include bleeding, mucosal damage to the blood vessels and digestive tract, lymphoid organ damage, amongst other lesions [1]. This infection was first reported in the Netherlands in 1923; it was subsequently reported in a number of countries that raise ducks [2]. As a result of its wide geographical distribution, rapid spread and high morbidity and mortality rates DEV has had a significant impact on the waterfowl industry on a global scale. However, the relative lack of molecular biology information on DEV, including information on its genome and proteome has restricted the identification of the pathogenic mechanism of this disease. This has hampered progress on developing a means to prevent and control this infection.

Autophagy is a widely conserved mechanism in eukaryotic cells, it maintains the environmental stability in cells by targeting damaged organelles and exogenous proteins and sending them to the lysosome for degradation [3, 4]. During autophagy, a cell can form a double-membraned structure named an autophagosome, which engulfs damaged organelles and other proteins and then merges with a lysosome to form an autolysosome, which will eventually degrade the contents [3]. It was recently described that, during infection with different viruses, autophagy may play different roles within a cell, some in the prevention and others in the promotion of viral replication.

Several signaling pathways regulating cell autophagy have been reported [5–7]. The endoplasmic reticulum (ER) stress pathway is one such pathway, which has been widely studied. ER stress is a protective stress response in eukaryotic cells; when the mass of unfolded protein in a cell surpasses the protein-folding capacity of the ER, the stress response is triggered [8]. Infection in most cells has been found to cause stress [9] because of the large amount of viral proteins that need to be synthesized in the ER. Viral induction of the ER stress response has been examined in a number of experiments, where the expression of several indicator proteins was monitored. These proteins include glucose-regulated protein 78 (GRP78), glucose-regulated protein 94 (GRP94), and calreticulin [10, 11]. Encephalomyocarditis virus 2C and 3D protein increased the expression of GRP78, GRP94 and calreticulin inducing ER stress [12]. Dengue virus and West Nile virus (WNV) were found to induce ER stress through increased GRP78 expression [13, 14]. To maintain ER homeostasis, cells have evolved an adaptive response called the unfolded protein response (UPR). Three independent signaling pathways, PKR-like ER protein kinase (PERK), activating transcription factor 6 (ATF6), and inositol-requiring enzyme 1 (IRE1), regulate the UPR. Cells infected with some viruses, including Newcastle disease virus (NDV), Bluetongue virus and Varicella-zoster virus, have been shown to induce one or more branches of the UPR [15–17].

Although the functions of autophagy and the UPR are independent, increasing evidence suggests that these processes are closely related [18–20]. In addition, some reports have indicated that virus-triggered ER stress subsequently induces autophagy. Autophagy has been associated with the replication and pathogenic mechanism of several pathogens including Rotavirus [21] and NDV [15]. Our previous studies demonstrated that DEV can induce autophagy in duck embryo fibroblast (DEF) cells, which benefits its own replication [22]. However, the mechanism by which DEV induces autophagy remains unknown.

Here, we showed that DEV infection triggered ER stress and the UPR. This response included DEV-induced autophagy, which was primarily regulated through the PERK- eukaryotic initiation factor 2α (eIF2α) and IRE1-x-box protein1 (XBP1) pathways, and conversely the knockdown of PERK and IRE1 suppresses DEV replication.

## Materials and methods

### Cell, virus and plasmids

DEF cells were obtained from 9- to 11-day-old specific pathogen-free (SPF) duck embryos according to a previously published protocol [23] and cultured in Dulbecco’s modified Eagle’s medium (DMEM) (Gibco, Grand Island, NY, USA) supplemented with 5% fetal bovine serum (Gibco) and antibiotics (0.1 mg/ml streptomycin and 0.1 mg/ml penicillin) at 37°C in 5% CO_2_.

The DEV CSC strain was from the Chinese Institute of Veterinary Drug Control. Mouse monoclonal antibody against glycoprotein B (gB) was maintained in our laboratory, and produced by Genscript biotechnology limited company. To construct the GFP-RFP-LC3 recombination plasmid, the p-GFP-RFP plasmid was prepared in our laboratory. The duck *LC3B* gene was amplified from DEF cells with primers (LC3F: ATGCAACCGCCTCTG; LC3R: TCGCGTTGGAAGGCAAATC) designed according to the GenBank sequence of duck LC3B (NW_004676873.1). Then, duck LC3B was cloned into the plasmid using BamH I and Xho I restriction enzyme sites [22].

### Antibodies and reagents

Anti-phospho-PERK antibody was obtained from Pierce (Rockford, IL, USA). The antibody for phospho-eIF2α was obtained from Cell Signaling Technology (Beverly, MA, USA). Rabbit anti-LC3B antibody, mouse anti-β-actin antibody, rabbit anti-GRP78 antibody, rabbit anti-ATF6 antibody, rabbit anti-ATF4 antibody, and rabbit anti-phospho-IRE1 antibody were purchased from Sigma-Aldrich (St. Louis, MO, USA). Small interfering RNAs (siRNAs) targeting PERK and IRE1 were synthesized by Shanghai GenePharma Co. Ltd. WonderOrange ProteinQuantitationKit(US Everbright Inc, Suzhou,China).

### Virus infection and cell viability

DEF cells in 6-well plates were infected with DEV at a multiplicity of infection (MOI) of 1, and the supernatant was removed after absorption for 2 h in DMEM without any FBS. The cell monolayers were rinsed three times with sterile phosphate buffered saline (PBS, pH 7.2) and incubated in fresh complete medium at 37°C. DEF cells were washed three times with sterile PBS and then maintained in 2% FBS in the culture medium for various durations until the samples were harvested.

Then, DEF cells were infected according to the above methods, using double-distilled H2O (ddH2O) or dimethylsulfoxide (DMSO) as a control. A siRNA toxicity test was performed according to the instructions of the WST-1 Cell Proliferation and Cytotoxicity kit (Beyotime, Jiangsu, China). At the indicated times, DEF cells were collected for subsequent analysis [22].

### SDS-PAGE and western blot

Both virus-infected cells and siRNA-treated cells were collected as follows: cell protein was extracted using immunoprecipitation (IP) lysis buffer (Beyotime) with phenylmethylsulfonyl fluoride (PMSF) protease inhibitor (Beyotime). Protein samples were mixed with 5× loading buffer, boiled for 10 minutes, analyzed on 12% sodium dodecyl sulfate-polyacrylamide gels (SDS-PAGE) and transferred onto nitrocellulose membranes (GE Healthcare Life Science, Munich, Germany) according to the manufacturer’s instructions. The membranes were blocked with 3% bovine serum albumin (BSA) (Sigma) for 2 hours at room temperature and then incubated with primary antibody for 2 hours at room temperature. Then, the membranes were inoculated with IRDye 800 CW goat anti-mouse IgG or goat anti-rabbit IgG as secondary antibodies for 1 hour at room temperature. Detection was carried out using an Odyssey Infrared Fluorescence Scanning Imaging System (LI-COR Biosciences, Lincoln, NE, USA).

### Transmission electron microscopy

Transmission electron microscopy (TEM) observation of autophagy was carried out as previously described [22, 24]. DEF cells were grown in 25 cm^2^ plates and collected 24 hours post-infection with DEV, using mock infected cells as a control. Ultrathin sections were observed under an H-7650 transmission electron microscope (Hitachi, Tokyo,Japan). In the cytoplasm, the autophagosome-like vesicles with single or double membranes and diameters of 0.3–2.0 μm were clearly visible. The number of autophagosomes in DEV-infected and mock cells was counted.

Immunoelectron microscopy observation of autophagy also was carried out as previously described [25]. First antibodies were incubated for 1h at room temperature and washed five times in PBS for 5 min. The secondary antibodies were conjugated to colloidal gold, then incubated for 1h at room temperature, and washed five times. The sections were stained with 3% uranyl acetate for 10 min at room and observed as described above.

### RNA interference of PERK and IRE1

To further study the effects of UPR on viral replication, we designed and administered specific siRNA constructs against PERK and IRE1. The sequences of the siRNAs for PERK were as follows: AAGAGGACCTTGTGGAAGCTG (sense) and AAGGTCTCTAGTAATTATCAG (antisense). The sequences of the siRNAs for IRE1 were as follows: AAGACCGGCAGTTTCAGTACA (sense) and AAGCAGGATATTTGGTACGTG (antisense). Six-well plates were transfected with siRNA and Negative Control RNA for 24 hours and then infected with DEV. Cell samples were collected to determine the silencing effects and viral titers.

### XBP1 mRNA splicing by IRE1

Total RNA from DEV-infected or mock-infected DEF cells was isolated using TRIzol Reagent (Sigma). Two micrograms of RNA was reverse transcribed using a GoldScript cDNA Synthesis kit (Life Technologies, Carlsbad, CA, USA) with random primers (TaKaRa, DaLian, China). The XBP1 gene was amplified by RT-PCR with the forward primer 5′-AGAAGACGTGCAGCCTTTCC-3′ and the reverse primer 5′- TGCCCATTTTAACAGGAATCTCCA-3′. The PCR products were further digested with the restriction enzyme PstI (Thermo Fisher Scientific, Waltham, MA, USA) and then separated on a 1% agarose gel. As an internal control, DNA complementary to the GAPDH messenger RNA (mRNA) was also amplified using the forward primer 5′-AGATGCTGGTGCTGAATACG-3′ and the reverse primer 5′ -CGGAGATGATGACACGCTTA-3′.

### TCID_50_

DEF cell monolayers were infected with DEV that had been serially diluted from 10^−1^–10^−8^ in 96-well plates. The virus was removed after incubation for 2 hours at 37°C, and the cells were washed three times with sterile PBS. DEF cells were maintained in 2% FBS culture medium at 37°C for a further 72 hours, cellular pathological changes were observed and recorded. Viral titers were determined according to the Reed-Muench method [22].

### Statistical analysis

All experimental results are expressed as the mean ± standard deviation (SD). All data were analyzed in three independent experiments. Tukey’s test was used for statistical analysis. The difference between two group means is presented as p < 0.05 (*) and p < 0.01 (**).

## Results

### Notable autophagy and ER stress were triggered by DEV infection

**Fig 1:**
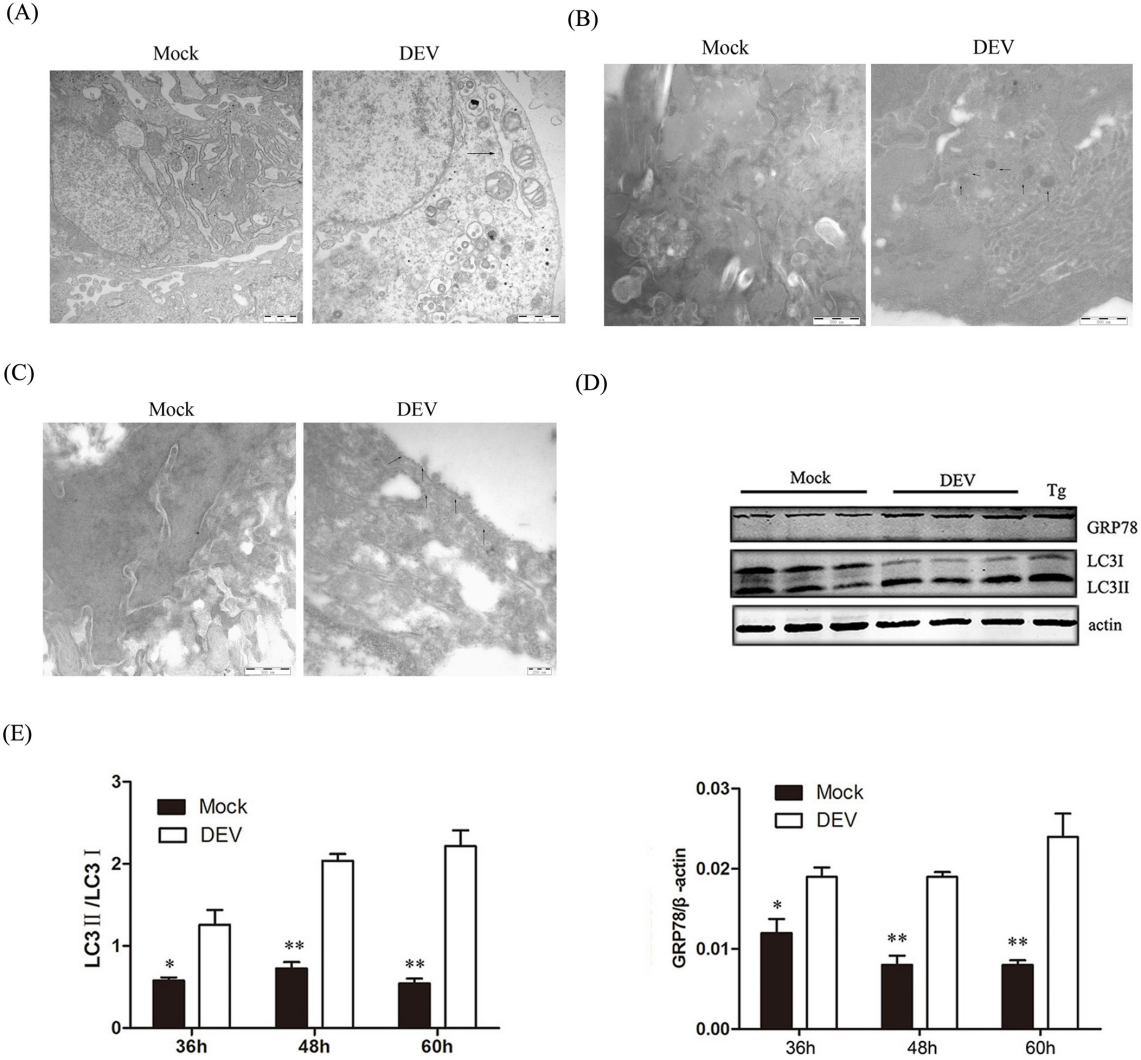
ER stress are activated in DEF cells upon DEV infection. (A)The ER ultrastructure in DEV-infected DEF cells under electron microscopy.Untreated cells were used as negative controls. Black arrows indicate the ER. Dilated ERs exist in DEV-treated cells. Scale bars: 1 μm. (B) DEF cells were infected with DEV at an MOI of 1 for 36h. Autolysosomes labeled with p62 antibodies were observed by immunoelectron microscopy. The arrow indicates colloidal gold dots representing p62. Scale bar: 500 nm. Untreated cells were used as negative controls. (C)DEF cells were infected with DEV at an MOI of 1 for 36 h. Endoplasmic reticulums labeled with GRP78 antibodies were observed by immunoelectron microscopy. The arrow indicates colloidal gold dots representing GRP78. Scale bar: 500 nm. Untreated cells were used as negative controls. (D) DEF cells were mock-treated or infected with DEV at a MOI of 1 and collected at 36, 48 and 60 hours post-infection and subjected to a western blot with antibodies against GRP78, LC3, β-actin (loading control), or DEV gB protein, as indicated. Thapsigargin (Tg) as a ER inducer. (E) Intensity band ratio of LC3-II to LC3-I and GRP78 to β-actin.

It is well known that many viral infections involve autophagy. We have demonstrated that DEV infection can induce autophagy, We tested whether DEV could induce ER stress and subsequently regulate autophagy. ER expansion was also observed under TEM in DEV-infected cells compared with the mock group (Fig 1A). ER expansion and abnormal ER morphology suggested that stress could alter ER homeostasis. We measured the expression of the ER stress marker protein GRP78 to further verify that ER stress was induced by DEV. GRP78 was significantly up-regulated after DEV infection compared with uninfected cells (Fig 1B), which suggested that ER stress was triggered by DEV infection.

### PERK and IRE1 pathways was activated under ER stress and was involved in DEV-induced autophagy

**Fig 2:**
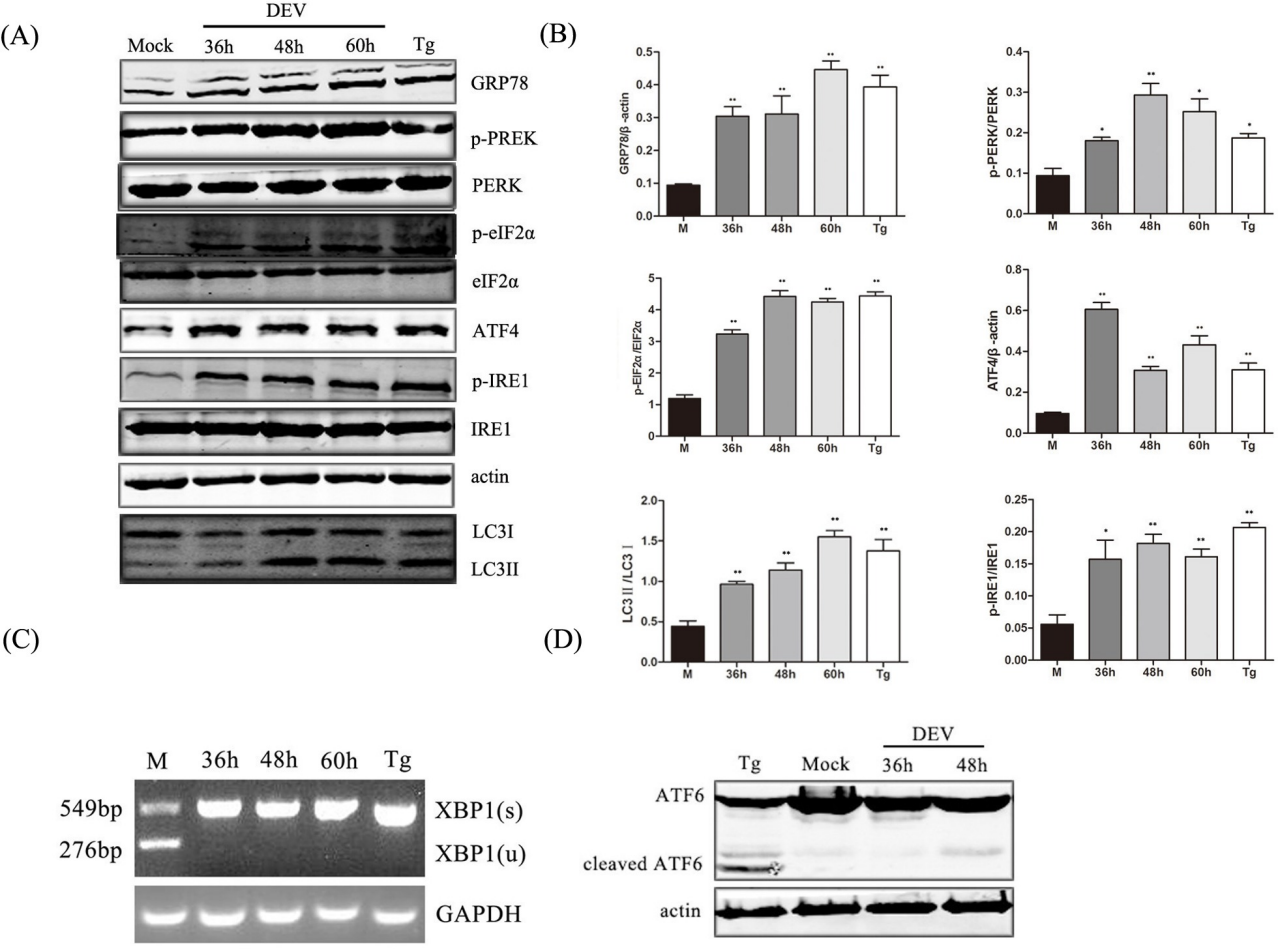
PERK and IRE1 pathways was activated under ER stress and was involved in DEV-induced autophagy. (A) DEF cells were infected with DEV at a MOI of 1 or treated with 300 nM Tg for 24 hours, and whole-cell protein extracts were collected at 36, 48, and 60 hours post-infection and then subjected to immunoblotting analysis of GRP78, p-PERK, p-eIF2α, LC3, ATF4, p-IRE1 and β-actin using the indicated antibodies. Tg is an inducer of ER stress. (B) Ratios of GRP78 to β-actin, p-PERK to PERK, p-eIF2α to eIF2α, ATF4 to actin, and p-IRE1 to IRE1, and LC3-II to LC3-I. (C) Activation of the IRE1-XBP1 signaling pathway. Total RNA was isolated from DEF cells, and RT-PCR was performed using XBP1-specific primers. PCR products were digested with PstI. Spliced XBP1 (s) products, 549 bp; Unspliced XBP1 (u) products, 276 bp; GAPDH was used as the loading control. (D) Inactivation of the ATF6 signaling pathway. DEF cells were infected with DEV at a MOI of 1 or treated with 300 nM Tg for 24 hours, and whole-cell protein extracts were collected at 36, 48, and 60 hours post-infection and then subjected to immunoblotting analysis of ATF6 and β-actin using the indicated antibodies.

We aimed to determine which sensor proteins, PERK, IRE1 or ATF6, activate the UPR in response to ER stress. PERK and eIF2α were significantly phosphorylated in DEV-infected cells. The abundance of ATF4, an effector of PERK and eIF2α, also increased, which indicated that the PERK-eIF2α pathway was activated under DEV-induced ER stress (Fig 2A and 2B).

Phosphorylation of IRE1 activated its ribonuclease enzymes to shear X-box protein 1 (XBP1) mRNA by removing 26-nt gene introns and moved spliced XBP1 (XBP1s) to within the nucleus.This led to the expression UPR-related genes, including ER stress chaperone proteins and ER degradation pathway-related proteins. First, expression of the Phosphorylation IRE1 was evaluated by western blot and found to be up-regulated following infection. Expression of XBP1s and unspliced XBP1 mRNA were measured by semi-quantitative PCR. XBP1s mRNA was detected in DEV-infected DEF cells at 36 hours post-infection, while unspliced XBP1 mRNA was found in mock infected cells at the same point (Fig 2A, 2B and 2C), which indicated that DEV infection activated the IRE1-XBP1 pathway under ER stress.

When the ATF6 pathway is activated, ATF6 translocates from the ER to the Golgi apparatus and is cleaved by trans-membrane proteases, releasing the active N-terminal 50-kDa ATF6. In this study, DEV infection did not increase the degradation of the 90-kDa ATF6 precursor to yield a 50-kDa cleavage product in DEF cells (Fig 2D). These results indicate that DEF infection cannot activate the ATF6 pathway of the UPR.

### Inhibition the PERK-eIF2α and IRE1-XBP1 pathways suppressed DEV replication

**Fig 3:**
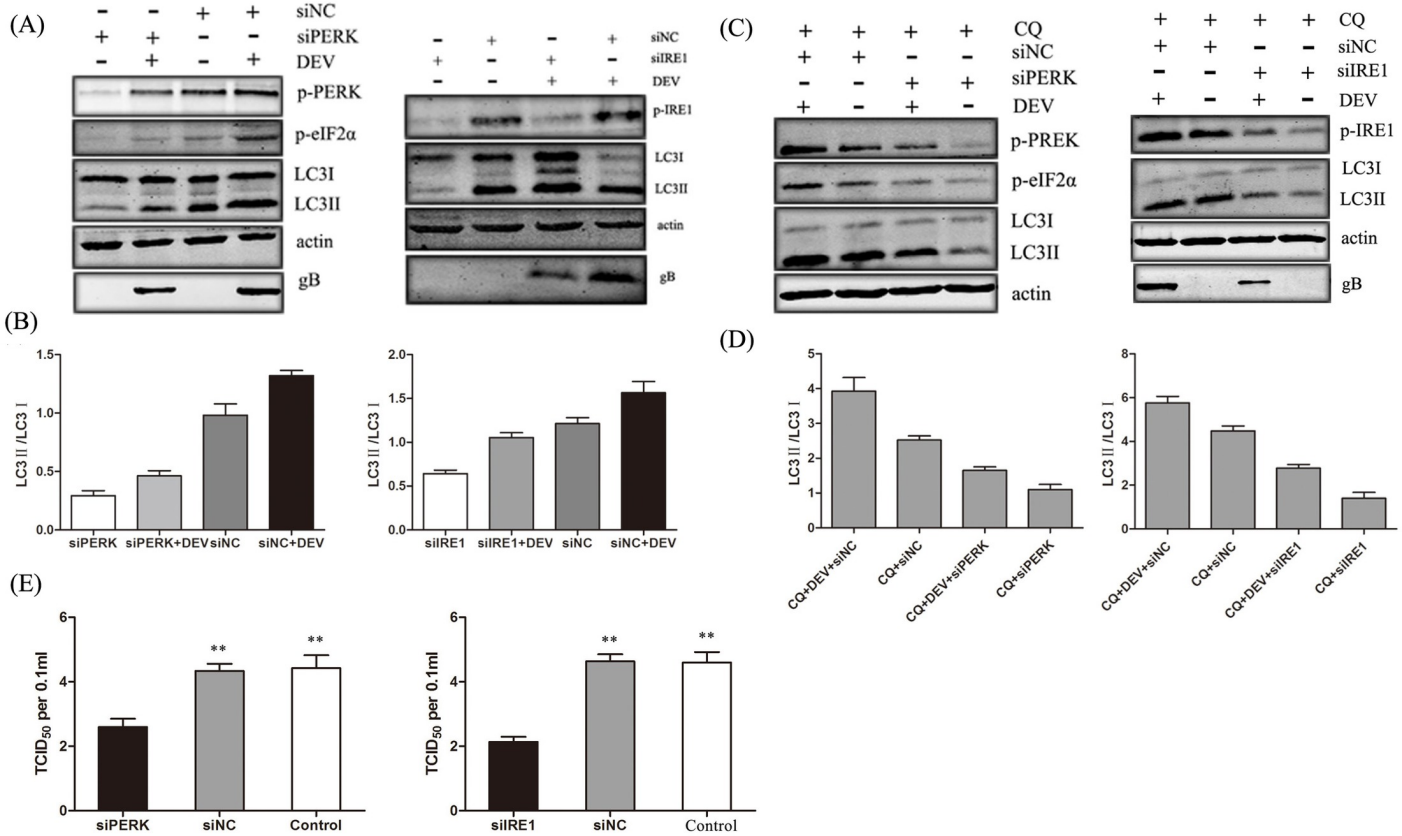
Effects of PERK-eIF2α and IRE1-XBP1 pathways on DEV replication. (A) DEF cells were transfected with a Negative Control siRNA (siNC) or siRNAs directed against PERK or IRE1 (100 pmol/ml), followed by DEV infection. Whole-cell protein extracts were collected at 48 hours post-infection and then subjected to immunoblotting analysis of PREK, p-PERK, eIF2α p-eIF2α, IRE1, p-IRE1, LC3, and β-actin using the indicated antibodies. (B) Ratio data of LC3-II to LC3-I in treated DEF cells. (C) CQ was added to each sample in presented treated DEF cells.Whole-cell protein extracts were collected at 48 hours post-infection and then subjected to immunoblotting analysis of PREK, p-PERK, eIF2α p-eIF2α, IRE1, p-IRE1, LC3, and β-actin using the indicated antibodies. (D) Ratio data of LC3-II to LC3-I in treated DEF cells. (E) The virus yields were determined at 48 hours post-infection and expressed as TCID_50_ per 0.1 ml.

We next tried to determine the roles of the PERK-eIF2α and IRE1-XBP1 pathways in DEV replication. siRNAs were synthesized to reduce the expression of PERK and IRE1. After siRNA treatment endogenous PERK, eIF2α and IRE1 were not detected by western blot and repressed the conversion of LC3-I to LC3-II in DEV-infected cells. Less DEV gB protein was observed in PERK- and IRE1-knockdown cells compared with control cells (Fig 3A and 3B). Chloroquine (CQ), as a autophagy inhibitor by inhibiting lysosomal activity at a late stage, was added to each sample presented, to accurately measure the autophagy flux in each condition.These data showed consistent with above results(Fig 3C and 3D). In addition, DEV titers were significantly reduced in PERK- and IRE1-knockdown cells compared with control cells. TCID_50_ was used to measure the viral titers (Fig 3E). Taken together, these data also indicate that the PERK and IRE1 pathways of the UPR are involved in DEV-induced autophagy and that the inhibition of ER stress suppresses DEV replication.

### Cell viability was unaffected by RNA interference and drugs

**Fig 4:**
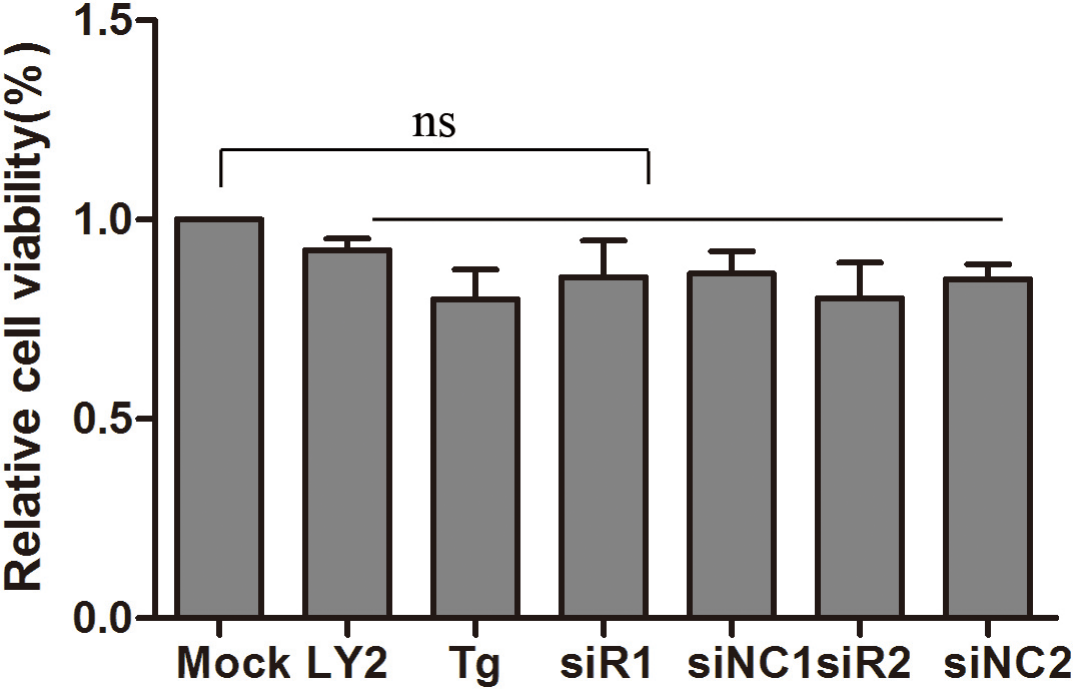
siRNAs or drugs had no effect on cell viability. siRNA and NC-siRNA were transfected or drugs treated for 48 hours, cell viability was tested using a WST-1 Cell Proliferation and Cytotoxicity kit, and absorbent density at 450 nm was expressed as relative cell viability.

siRNAs or drugs might influence cell viability and thus our results. The effects of the compounds used in this study on cell viability were detected by a WST-1 Cell Proliferation and Cytotoxicity kit. The viability of treated cells was almost equal to that of mock cells. Therefore, these siRNAs and drugs did not effect DEF cell viability (Fig 4).

## Discussion

Autophagy is an important cellular adaptive process that maintains cellular homeostasis. To date, several viruses have been shown to induce an autophagic response in infected cells; this response is closely linked to the propagation of these viruses [15, 16]. We previously found that DEV triggered autophagy in host cells for its own benefit. However, until now, the mechanism of this induction was unknown. In this study, we present further evidence that ER stress, in addition to the autophagic response, was triggered by DEV infection. Prior to this study it was unclear whether there was a link between ER stress and autophagy during DEV infection. Our research suggests that ER stress may be involved in the regulation of autophagy induced by DEV infection.

ER is an important multifunctional organelle; for several viruses, the ER is the site of viral replication and maturation [26]. In the process of infection, which involves a large amount of viral protein synthesis, unfolded or misfolded proteins induce the ER stress response and lead to the activation of UPR. In our study, we used western blot to show that GRP78 is significantly up-regulated in DEV-infected cells, which suggested that the ER stress was activated. The link between this protein, the UPR and autophagy must be explored further in future studies.

PERK dimerization and trans-autophosphorylation leads to the activation of eIF2α by phosphorylation at Ser51. PERK and eIF2α are the signaling pathways involved in the early stage of ER stress [27]. Some reports have reported an association between the PERK pathway and autophagy and that phosphorylation of the downstream regulator eIF2α plays an important role in ER stress-induced autophagy [28]. Therefore, we explored whether the PERK-eIF2α pathway participates in the regulation of ER stress during DEV infection-induced autophagy. In our study, phosphorylation of both PERK and eIF2α was up-regulated, which is consistent with the increased expression of the ER stress marker gene GRP78 and autophagy marker gene LC3-II. In addition, knockdown of PERK by siRNA decreased the level of DEV-induced autophagy and virus production. These results showed that ER stress was involved in DEV-induced autophagy via the PERK-eIF2α pathway.

Under ER stress, IRE1-XBP1 activation can lead to JNK signaling, which is necessary for an autophagosome to form [29]. An IRE1 signaling pathway inhibitor significantly decreased viral replication, indicating that IRE1 activation is essential for HCV infection and autophagosome formation [30]. In this study, the IRE1 pathway protein p-IRE1 was up-regulated, and spliced XBP1 mRNA was detected and no unspliced XBP1 was found in DEV-infected DEF cells. In addition, knockdown of IRE1 by siRNA decreased the level of DEV-induced autophagy and virus production. These results revealed that the IRE1-XBP1 pathway was activated during DEV infection-induced autophagy.

ATF6 is a type II ER transmembrane protein that contains a basic region/leucine zipper (bZIP) domain in the cytosol and a stress-sensing domain in the ER lumen [29]. Several different viruses have been demonstrated to activate the ATF6 pathway, promoting replication, including WNV, NDV and African swine fever virus (ASFV). In cells infected with WNV, ATF6 is activated and promotes viral replication by inhibiting the signal transduction and late stage interferon (IFN) signals [31]. In this study, although expression of ATF6 could be observed by western blot, cleaved ATF6 was not found, which indicates that the ATF6 pathway does not trigger DEV-induced autophagy.

In conclusion, our study indicates a novel mechanism of DEV infection-induced autophagy mediated by ER stress. In addition, our results suggest that autophagy induction involves PERK and IRE1 of the UPR and that interference reducing activity in these two pathways can suppress DEV replication. These data lay the foundation for future studies to better understand the molecular mechanisms of autophagy in relation to DEV infection.
